# Childhood victimization and inflammation in young adulthood: A genetically sensitive cohort study

**DOI:** 10.1016/j.bbi.2017.08.025

**Published:** 2018-01

**Authors:** Jessie R. Baldwin, Louise Arseneault, Avshalom Caspi, Helen L. Fisher, Terrie E. Moffitt, Candice L. Odgers, Carmine Pariante, Antony Ambler, Rosamund Dove, Agnieszka Kepa, Timothy Matthews, Anne Menard, Karen Sugden, Benjamin Williams, Andrea Danese

**Affiliations:** a MRC Social, Genetic and Developmental Psychiatry Centre, Institute of Psychiatry, Psychology and Neuroscience, King’s College London, London, UK; b Department of Psychology and Neuroscience, Duke University, Durham, NC, USA; c Department of Psychiatry and Behavioral Sciences, Duke University, Durham, NC, USA; d Department of Psychology and Social Behavior, University of California, Irvine, Irvine, CA, USA; e Sanford School of Public Policy, Duke University, Durham, NC, USA; f Department of Psychological Medicine, Institute of Psychiatry, Psychology and Neuroscience, King’s College London, London, UK; g Department of Child and Adolescent Psychiatry, Institute of Psychiatry, Psychology and Neuroscience, King’s College London, London, UK; h National and Specialist CAMHS Trauma and Anxiety Clinic, South London and Maudsley NHS Foundation Trust, London, UK

**Keywords:** Childhood victimization, Childhood maltreatment, Bullying victimization, Early life stress, Inflammation, C-reactive protein, Sex differences

## Abstract

**Objective:**

Childhood victimization is an important risk factor for later immune-related disorders. Previous evidence has demonstrated that childhood victimization is associated with elevated levels of inflammation biomarkers measured decades after exposure. However, it is unclear whether this association is (1) already detectable in young people, (2) different in males and females, and (3) confounded by genetic liability to inflammation. Here we sought to address these questions.

**Method:**

Participants were 2232 children followed from birth to age 18 years as part of the Environmental Risk (E-Risk) Longitudinal Twin Study. Childhood victimization was measured prospectively from birth to age 12 years. Inflammation was measured through C-reactive protein (CRP) levels in dried blood spots at age 18 years. Latent genetic liability for high inflammation levels was assessed through a twin-based method.

**Results:**

Greater exposure to childhood victimization was associated with higher CRP levels at age 18 (serum-equivalent means were 0.65 in non-victimized Study members, 0.74 in those exposed to one victimization type, and 0.81 in those exposed to poly-victimization; p = 0.018). However, this association was driven by a significant association in females (serum-equivalent means were 0.75 in non-victimized females, 0.87 in those exposed to one type of victimization, and 1.19 in those exposed to poly-victimization; p = 0.010), while no significant association was observed in males (p = 0.19). Victimized females showed elevated CRP levels independent of latent genetic influence, as well as childhood socioeconomic status, and waist-hip ratio and body temperature at the time of CRP assessment.

**Conclusion:**

Childhood victimization is associated with elevated CRP levels in young women, independent of latent genetic influences and other key risk factors. These results strengthen causal inference about the effects of childhood victimization on inflammation levels in females by accounting for potential genetic confounding.

## Highlights

• Childhood victimization predicted elevated levels of CRP at age 18.
• The association between child victimization and CRP levels was specific to females.
• Latent genetic influences on CRP levels did not explain the association in females.

## 1 Introduction

Childhood victimization is a major risk factor for a range of mental and physical health problems in later life (Varese et al., 2012, Danese and Tan, 2014, Takizawa et al., 2014, Takizawa et al., 2015). In order to assist victimized children and to prevent later illness, it is important to identify the biological mechanisms through which victimization influences health outcomes later in life. Because many disorders linked to child victimization (e.g., depression, post-traumatic stress disorder, psychosis, obesity, and cardiovascular disease) have pro-inflammatory origins (Miller et al., 2009, Danese and Baldwin, 2017), inflammation has been proposed as a mechanism through which victimization may become ‘biologically embedded’ (Danese and McEwen, 2012).

Children who experience victimization show elevated levels of inflammation biomarkers several decades later. For example, a previous study from our team found that maltreated children in the Dunedin Study showed an elevated risk of clinically relevant C-reactive protein (CRP) and other inflammation biomarkers at age 32 (Danese et al., 2007), independent of other key risk factors in childhood and adulthood. Furthermore, children exposed to peer bullying victimization in the 1958 British National Child Development Study showed elevated CRP levels at age 45, after accounting for other potential confounders in childhood and adulthood (Takizawa et al., 2015). Finally, a meta-analysis of 25 studies showed that different types of childhood trauma were associated with elevated levels of CRP and other inflammation biomarkers at a mean age of 42 years (Baumeister et al., 2015). Although the association between childhood victimization and inflammation in later adulthood is now well established, key developmental questions relating to onset, sex differences, and genetic influences remain unanswered.

With regard to onset, it is unclear whether victimized children only show high inflammation levels decades after exposure, or whether these effects can be detected earlier in life. Research in this area has mainly included participants at midlife (Baumeister et al., 2015) and findings from studies of young people are inconsistent. For example, one study of young adults showed a positive association between childhood victimization and inflammation (Copeland et al., 2014), while other studies of children and adolescents showed conditional associations (Danese et al., 2011, Miller and Cole, 2012) or an inconsistent association according to the measure of victimization (Slopen et al., 2013). A better understanding of the emergence of the association between childhood victimization and inflammation is important to guide the timing of preventative interventions for victimized children.

With regard to sex differences, it is unclear whether victimized boys and girls differ in their liability to high inflammation levels. Experimental studies in animal models have suggested sex-specific effects of early life stress on inflammation (Ganguly and Brenhouse, 2015), and findings in humans indicated that females may be more vulnerable than males to high inflammation levels in response to acute stress (Rohleder et al., 2001) and in the context of socioeconomic disadvantage (Steptoe et al., 2002, Rathmann et al., 2006, Howe et al., 2010). Furthermore, victimized women might be more vulnerable than men to other cardio-metabolic outcomes correlated with inflammation (Danese and Tan, 2014, Murphy and Loria, 2017). However, most previous studies testing the association between childhood victimization and inflammation in young people did not examine sex differences.

With regard to genetic influences, it is unclear whether high inflammation levels in victimized children reflect environmental effects of victimization, or the result of genetic liability. Plasma levels of inflammation biomarkers, such as CRP, are moderately heritable (e.g., 40%; Pankow et al., 2001), and inflammatory genes might influence children’s risk of experiencing victimization through gene-environment correlation (Jaffee and Price, 2007, Danese and Baldwin, 2017). For example, genetic pathways related to the immune system predict risk for several psychiatric diagnoses (The Network and Pathway Analysis Subgroup of the Psychiatric Genomics Consortium, 2015). In turn, early expressions of the liability to these conditions, such as emotional dysregulation or oppositional behavior, might increase the risk of victimization in children (Danese and McCrory, 2015). Similarly, psychopathology and impaired emotion regulation in parents have been linked to high risk of victimization in their children (Danese and McCrory, 2015). These examples of evocative and passive gene-environment correlations illustrate alternative and yet untested mechanisms underlying the association between childhood victimization and inflammation.

To address these research gaps, we tested the association between childhood victimization and CRP levels in a population-representative sample of twins followed from birth to age 18. We hypothesized that victimized children would show a small elevation in CRP levels by age 18; that the association would be stronger in females; and that genetic influences would contribute to the association.

## 2 Method

### 2.1 Study sample

Participants were members of the Environmental Risk (E-Risk) Longitudinal Twin Study, which tracks the development of a birth cohort of 2232 British children. The sample was drawn from a larger birth register of twins born in England and Wales in 1994–95 (Trouton et al., 2002). Full details about the sample are reported elsewhere (Moffitt and E-Risk Study Team, 2002). Briefly, the E-Risk sample was constructed in 1999–2000, when 1116 families (93% of those eligible) with same-sex 5-year-old twins participated in home-visit assessments. This sample comprised 56% monozygotic (MZ) and 44% dizygotic (DZ) twin pairs; sex was evenly distributed within zygosity (49% male). Families were recruited to represent the U.K. population of families with newborns in the 1990s, on the basis of residential location throughout England and Wales and mother’s age. Teenaged mothers with twins were over-selected to replace high-risk families who were selectively lost to the register through non-response. Older mothers having twins via assisted reproduction were under-selected to avoid an excess of well-educated older mothers. The study sample represents the full range of socioeconomic conditions in Great Britain, as reflected in the families’ distribution on a neighborhood-level socioeconomic index (ACORN [A Classification of Residential Neighbourhoods], developed by CACI Inc. for commercial use in Great Britain) (Odgers et al., 2012): 25.6% of E-Risk families live in “wealthy achiever” neighborhoods compared to 25.3% nationwide; 5.3% vs. 11.6% live in “urban prosperity” neighborhoods; 29.6% vs. 26.9% live in “comfortably off” neighborhoods; 13.4% vs. 13.9% live in “moderate means” neighborhoods; and 26.1% vs. 20.7% live in “hard-pressed” neighborhoods. E-Risk underrepresents “urban prosperity” neighborhoods because such households are likely to be childless.

Follow-up home visits were conducted when the children were aged 7 (98% participation), 10 (96% participation), 12 (96% participation), and, most recently in 2012–2014, at 18 years (93% participation). Home visits at ages 5, 7, 10, and 12 years included assessments with participants as well as their mother (or primary caretaker); the home visit at age 18 included interviews only with the participants. Each twin participant was assessed by a different interviewer. The average age of the twins at the time of the assessment was 18.4 years (*SD* = 0.36); all interviews were conducted after the 18^th^ birthday. There were no differences between those who did and did not take part at age 18 in terms of socioeconomic status (SES) assessed when the cohort was initially defined (*Χ^2^* = 0.86, *p* = 0.65), age-5 IQ scores (*t* = 0.98, *p* = 0.33), age-5 internalizing or externalizing behavior problems (*t* = 0.40, *p* = 0.69 and *t* = 0.41, *p* = 0.68, respectively), or childhood victimization (*z* = 0.51, *p* = 0.61).

The Joint South London and Maudsley and the Institute of Psychiatry Research Ethics Committee approved each phase of the study. Parents gave informed consent and twins gave assent between 5 and 12 years and then informed consent at age 18.

### 2.2 Childhood victimization

We have previously reported evidence on the reliability and validity of our measurement of childhood victimization (Danese et al., 2016) and full details of this measure are reported in the Supplementary Material. Exposure to several types of victimization was assessed repeatedly when the children were 5, 7, 10, and 12 years of age and dossiers were compiled for each child with cumulative information about exposure to domestic violence between the mother and her partner; frequent bullying by peers; physical maltreatment by an adult; sexual abuse; emotional abuse and neglect; and physical neglect. Following Finkelhor et al. (2007), we have operationalized childhood victimization as the total number of victimization types that a child experiences: 73.5% of children had experienced zero victimization types; 20.1% experienced 1 victimization type; 3.8% experienced 2 victimization types; 1.8% experienced 3 victimization types; 0.8% experienced 4 victimization types; and 0.1% experienced 5 victimization types. We winsorised the poly-victimization distribution into a 3-category variable including children who were not exposed to victimization, children who experienced one type of victimization, and children who experienced multiple types of victimization.

### 2.3 C-Reactive protein (CRP)

We collected capillary blood on filter paper during a home visit at the age-18 follow-up assessment in 1937 Study members (94% of those who took part in the assessment). Study members’ fingers were sterilised with an alcohol swab and incised with a retractable lancet (Bunzel Healthcare, catalogue number 366594). The first blood drop was discarded on tissue. Five subsequent drops of 50ul each were collected on a protein saver card (Fisher Scientific, catalogue number 10531018). The cards were placed in an air-tight drying box containing 2 Maxipax absorbent packets each containing 10 g silica gel and allowed to dry completely overnight. The cards were then moved into Ziplock bags with at least 2 absorbent packets per card and a humidity indicator card and refrigerated. The cards were transported to a central laboratory at the SGDP Centre for storage at −80 C within 2 weeks of collection. Cores taken from dried blood spots were eluted overnight on the day prior to laboratory analyses. The following morning, samples were shaken on a plate shaker for 30 min. CRP was quantified via Sandwich ELISA using the Human C-Reactive Protein ELISA Kit KHA0031 (Life Technologies, UK) according to manufacturers’ instructions. The kit has a lower limit of detection <10 pg/mL. Standard deviations were calculated from the samples' duplicates giving a coefficient of variation of 3.3%. Paired collection of dried blood spot and serum in n = 98 Study members was performed to derive a within-study conversion equation for CRP levels, where serum CRP value = 6.51 * (blood spot CRP value) + 0.14.

### 2.4 Latent genetic risk score

We estimated genetic influence on CRP levels through a latent score indexing twin-pair zygosity and the presence of high CRP at age 18 in the co-twin. This method has been widely used to account for additive genetic influences on a trait (Kendler et al., 1995, Jaffee et al., 2005, Van Lier et al., 2007, Arseneault et al., 2011, Wigman et al., 2011, Baldwin et al., 2016). We defined high CRP as the top tertile of the distribution, consistent with CDC/AHA guidelines for risk prediction in adults (Pearson et al., 2003). In order to build the latent genetic risk score, we considered that monozygotic twins share 100% of their genes while dizygotic (DZ) twins share on average 50% of their genes. Therefore, a child’s genetic liability was coded ‘lowest risk’ if they had a monozygotic co-twin without high CRP, ‘low risk’ if they had a dizygotic co-twin without high CRP, ‘high risk’ if they had a dizygotic co-twin with high CRP, and ‘highest risk’ if they had a monozygotic co-twin with high CRP. This method of estimating genetic risk for elevated CRP allowed us to account for a substantial proportion of variance in CRP levels, whilst retaining power by including the full sample of twins rather than restricting to twin pairs discordant for victimization.

### 2.5 Other risk factors for inflammation

#### 2.5.1 Childhood socioeconomic status

Socioeconomic disadvantage is associated with high CRP levels (Danese et al., 2009) and therefore we adjusted for childhood family socioeconomic status (SES). The family SES at the age of 5 years was defined through a standardized composite of parental income, education and occupation. The three SES indicators were highly correlated (r = 0.57–0.67) and loaded significantly onto one latent factor. The population-wide distribution of the resulting factor was divided in tertiles for analyses (Trzesniewski et al., 2006).

#### 2.5.2 Waist-hip ratio at age 18

Adipose tissue produces pro-inflammatory cytokines (Mohamed-Ali et al., 1997), and in particular, abdominal/central obesity – measured by waist-hip ratio - is correlated with high CRP levels over and beyond other measures of body mass (Visser et al., 1999, Brooks et al., 2010). We therefore adjusted for waist-hip ratio to account for the effect of abdominal obesity. Trained research workers measured waist and hip circumference of Study members at age 18, and waist-hip ratio was calculated by dividing waist circumference by hip circumference.

#### 2.5.3 Body temperature

Elevated body temperature is part of the physiological response to infections and injury (Merck, 2006). Therefore, we adjusted for body temperature to account for the possible effect of unrecognised infectious and inflammatory disorders on CRP levels (Danese et al., 2011). Trained research workers measured body temperature (by infrared digital ear thermometer Omron GT510) at the time of CRP assessment.

### 2.6 Statistical analysis

Of the 2066 children who participated in the E-Risk Study assessments at age 18 years, 1789 had complete data for childhood victimization, age-18 CRP levels and the latent genetic score. Study members with complete data did not differ from those with missing data with regard to childhood victimization (OR = 0.89, CI = 0.70–1.14, p = 0.37), gender (OR = 0.85, CI = 0.64–1.13, p = 0.26) or family SES (OR = 1.05, CI = 0.88–1.25, p = 0.57). Furthermore, we excluded 57 Study members with serum-equivalent CRP >10 mg/L as they were likely to have acute trauma, infections, or pathology (Pearson et al., 2003), giving us a final sample of N = 1732.

CRP was log-transformed to improve normality of its distribution. We used Generalized Estimating Equation (GEE) linear regression models in STATA 14 (College Station, TX) to predict CRP levels at age 18 and account for clustering of twins within families. First, we tested whether childhood victimization predicted elevated levels of CRP at age 18 years. Second, we stratified these analyses by gender to test sex-specific effects of childood victimization on CRP. Third, we tested the genetic contribution to CRP levels at age 18, by (i) running a univariate twin model in the R package ‘OpenMx’ to decompose the variation in CRP into additive genetic, shared environmental, and unique environmental components; and (ii) testing whether a latent genetic risk score predicted CRP levels at age 18. Fourth, we tested whether childhood victimization predicted elevated levels of CRP at age 18 years independent of the latent genetic risk score as well as other key risk factors for high CRP.

## 3 Results

### 3.1 Do victimized children have elevated CRP levels at age 18?

**Fig. 1:**
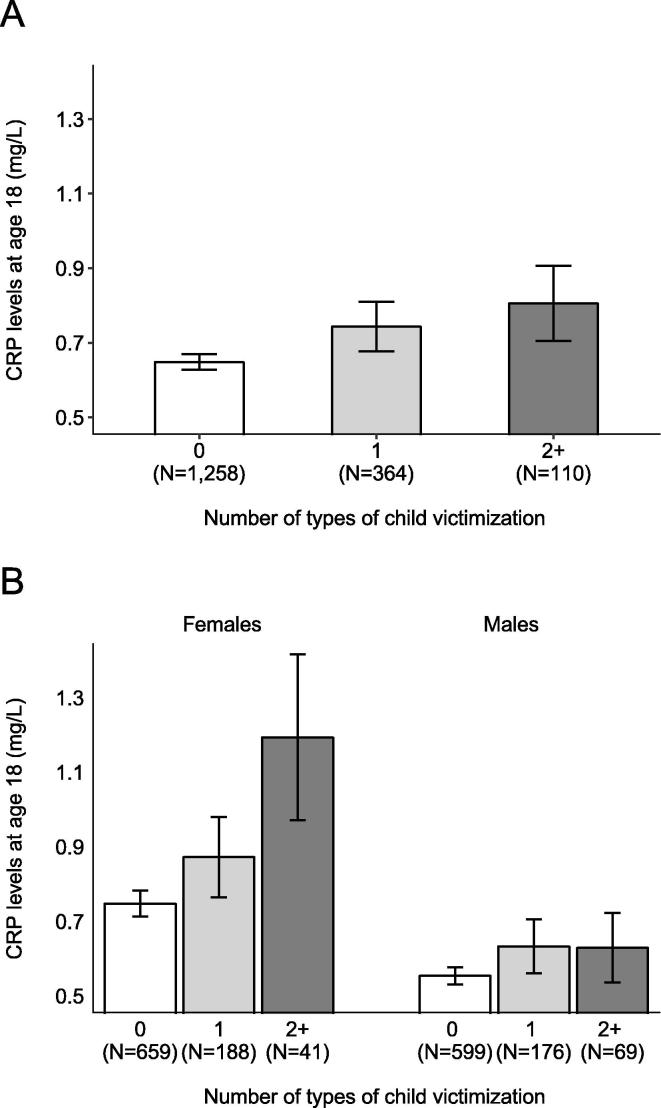
Association between childhood victimization and serum-equivalent CRP levels at age 18, in the overall sample (Panel A), and in analyses stratified by gender (Panel B). *Note*. Error bars show standard errors. Greater exposure to childhood victimization was associated with higher CRP levels at age 18 in the overall sample (b = 0.15, 95% CI = 0.03;0.27, p = 0.018) and in females (b = 0.25, 95% CI = 0.06;0.43, p = 0.010) but not in males (b = 0.10, 95% CI = −0.05;0.26, p = 0.19; sex-interaction p-value = 0.23).

We first tested the influence of childhood victimization on CRP levels at age 18 years (Fig. 1, Panel A). We found a positive linear association between exposure to childood victimization and CRP levels at age 18 (b = 0.15, 95% CI = 0.03; 0.27, p = 0.018). Compared to non-victimized children (N = 1258), children exposed to one type of victimization had higher CRP levels (N = 364; b = 0.18, 95% CI = 0.01; 0.36, p = 0.035) while the smaller group of children exposed to poly-victimization showed a nominal but statistically non-significant elevation in CRP levels (N = 110; b = 0.25, 95% CI = −0.05; 0.54, p = 0.10).

### 3.2 Do victimized males and females differ in their liability to elevated CRP levels at age 18?

Stratified analyses showed that the observed association between childhood victimization and elevated CRP levels at age 18 was driven by effects in female Study members (Fig. 1, Panel B). There was a positive linear association between childhood victimization and levels of CRP in females (b = 0.25, 95% CI = 0.06;0.43, p = 0.010). Relative to non-victimized girls, girls exposed to one type of victimization had a non-significant elevation in CRP levels (b = 0.21, 95% CI = −0.03;0.45, p=0.092) while poly-victimized girls showed elevated CRP levels (b = 0.56, 95% CI = 0.08;1.04, p = 0.021). However, no linear association was found in males (b = 0.10, 95% CI = −0.05; 0.26, p = 0.19). The sex-specific association was not explained by sex differences in exposure to victimization, as girls were not more likely than boys to experience victimization (Supplementary Table 1).

### 3.3 Is the latent genetic score a valid predictor of CRP levels at age 18?

**Fig. 2:**
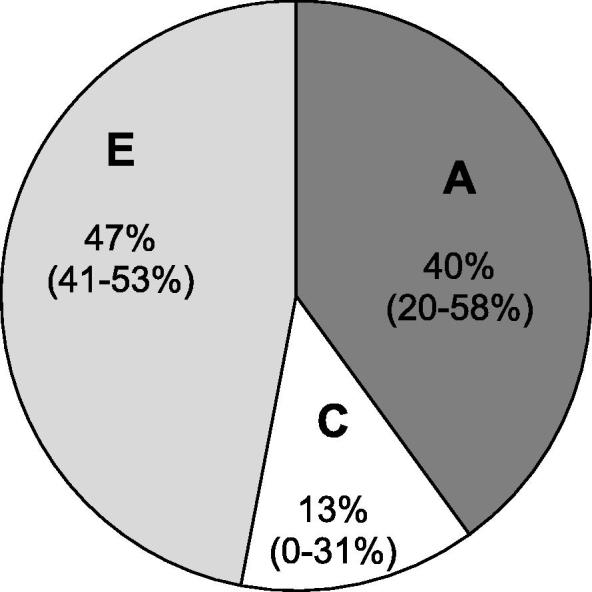
Pie chart showing the percentages of variance in CRP levels at age 18 accounted for by additive genetic influences (A), shared environmental influences (C) and unique environmental influences (E), with 95% confidence intervals. *Note*. Within-pair correlations for CRP levels were r = 0.56 for monozygotic twin pairs and r = 0.33 for dizygotic twin pairs.

**Fig. 3:**
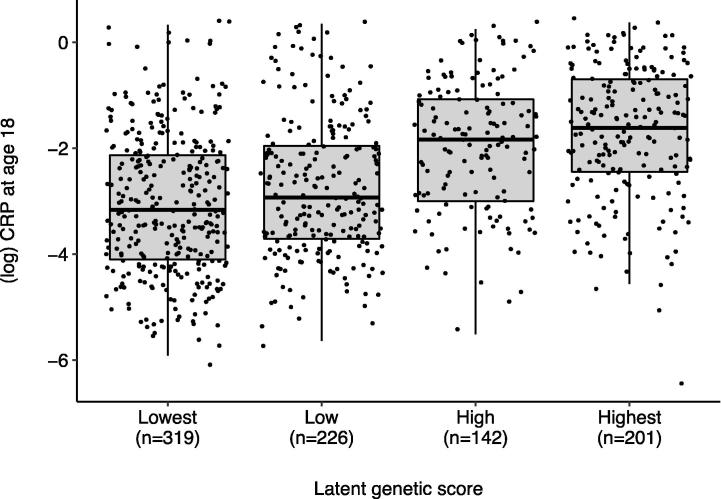
Association between latent genetic score and (log-transformed) CRP levels at age 18, in females. Note. Females with higher latent genetic scores showed higher levels of CRP at age 18 (beta = 0.35, CI = 0.28–0.42, p < 0.001; R^2^ = 15%).

We first tested if variation in CRP levels at age 18 years was genetically influenced in our sample in order to validate the latent genetic score for CRP (Jaffee et al., 2005). Using a univariate twin model, we found that the heritability of CRP levels was 40% (95% CI = 19%-58%) (Fig. 2). We then tested the association between the latent genetic score and CRP levels at age 18. The latent genetic score was associated with CRP levels, both in the overall E-Risk Study sample (n = 1732; beta = 0.33, p < 0.001; R^2^ = 13%) and in female Study members (n = 888; beta = 0.35, p < 0.001; R^2^ = 15%; Fig. 3). Because a statistically significant association between childhood victimization and CRP levels was found only in females, we hereafter focus our analyses in females.

### 3.4 Do victimized girls have elevated CRP levels at age 18 independent of their latent genetic risk?

To assess whether childhood victimization had an environmentally mediated influence on CRP levels in females at age 18, we tested whether victimized girls showed elevated CRP levels after accounting for their latent genetic scores. Poly-victimized girls showed elevated CRP levels at age 18 independent of their latent genetic score (Table 1, Model 1).

**Table 1:** Association between childhood victimization and (log-transformed) CRP levels in females at age 18. Results are presented as unstandardized coefficients and their 95% confidence intervals.

	Baseline Model	Model 1	Model 2	Model 3	Model 4
*Childhood victimization*					
None	Ref	Ref	Ref	Ref	Ref
1 type	0.21 (−0.03; 0.45)	0.06 (−0.13; 0.26)	0.17 (−0.07; 0.42)	0.16 (−0.08; 0.40)	0.04 (−0.16; 0.24)
2+ types	**0.56 (0.08; 1.04)**	**0.50 (0.12; 0.87)**	**0.52 (0.04; 1.00)**	**0.51 (0.04; 0.97)**	**0.44 (0.06; 0.82)**
*Latent genetic score*	**0.59 (0.52; 0.66)**	**0.59 (0.52; 0.66)**			**0.53 (0.46; 0.60)**

*Child socioeconomic status*					
High	Ref		Ref		Ref
Middle	0.09 (−0.19; 0.36)		0.07 (−0.21; 0.34)		−0.03 (−0.22; 0.16)
Low	**0.28 (0.00; 0.55)**		0.22 (−0.06; 0.50)		0.02 (−0.18; 0.22)
*Waist-hip ratio at age 18*	**3.82 (2.52; 5.11)**			**3.62 (2.32; 4.92)**	**3.46 (2.17; 4.74)**
*Body temperature*	**0.19 (0.04; 0.34)**			**0.18 (0.03; 0.32)**	**0.15 (0.01; 0.29)**

*Note*. The Baseline Model shows the bivariate associations between childhood victimization and risk factors for CRP levels with CRP levels. Model 1 shows the association between childhood victimization and CRP levels after adjusting for the latent genetic score. Model 2 shows the association between childhood victimization and CRP levels after adjusting for childhood socioeconomic status. Model 3 shows the association between childhood victimization and CRP levels after adjusting for waist-hip ratio and body temperature. Model 4 shows the association between childhood victimization and CRP levels after adjusting simultaneously for all covariates. N = 888 for all analyses. Missing values for waist-hip ratio (N = 25) and body temperature (N = 7) were replaced with the mean of the sample-wide distribution of available data. Numbers in bold represent statistically significant results.

### 3.5 Do victimized girls have elevated CRP levels at age 18 independent of key risk factors for elevated CRP?

We next considered that poly-victimized females may show elevated levels of CRP at age 18 due to other potential confounding factors and artefacts. We therefore tested whether the association was independent of family SES, and waist-hip ratio and body temperature at the time of CRP assessment, which were all associated with high levels of CRP in females (Table 1, Baseline Model). After accounting for family SES, poly-victimized girls still showed elevated levels of CRP at age 18 (Table 1, Model 2). After accounting for waist-hip ratio and body temperature, poly-victimized girls still showed higher levels of CRP at age 18 (Table 1, Model 3). Finally, after simultaneously accounting for family SES, waist-hip ratio, body temperature, as well as the latent genetic score, poly-victimized girls still showed higher levels of CRP at age 18 (Table 1, Model 4).

## 4 Discussion

In this prospective cohort study, we found that childhood victimization was associated with elevated levels of CRP at age 18. We observed a sex-specific effect, in that young women exposed to childhood poly-victimization had CRP levels that were 60% higher than non-victimized females, whereas no significant association was found in males. In females, this association was independent of latent genetic influence and other key risk factors for inflammation. Our findings are consistent with previous research demonstrating an association between childhood victimization and later elevated CRP levels (Danese et al., 2007, Copeland et al., 2014, Baumeister et al., 2015, Takizawa et al., 2015) and expand this literature in three ways.

First, because most previous research in this area included participants at midlife (Baumeister et al., 2015), we tested the association between childhood victimization and CRP levels at age 18 years to understand more about the emergence of this link. We observed that victimized children already show high CRP levels in young adulthood, consistent with findings from a previous epidemiological study (Copeland et al., 2014). This suggests that prevention of victimization or early interventions for victimized children may reduce inflammation in young adulthood and limit the associated health burden in later life (Shonkoff et al., 2009).

Second, building on suggestive evidence in animal models (Ganguly and Brenhouse, 2015) and human studies (Rohleder et al., 2001, Steptoe et al., 2002, Rathmann et al., 2006, Howe et al., 2010), we tested for sex-specific effects of early-life stress on inflammation. We found that the association between childhood victimization and CRP levels was present in young women, but not in men. Although a meta-analysis of studies with participants in midlife did not show moderation of the effects of childhood trauma on inflammation by gender (Baumeister et al., 2015), previous research in young people has not systematically tested for sex differences. It is possible that females could show greater vulnerability to the effects of stress on inflammation in early life, consistent with the sexual dimorphism in other stress-related systems and in the risk for emotional and cardiovascular disorders (Bale and Epperson, 2015, Murphy and Loria, 2017). Sex hormones may partly contribute to these effects, as increases in testosterone levels in male adolescents have been shown to negatively correlate with CRP levels (Shanahan et al., 2013). The non-significant association between childhood victimization and CRP levels observed in young men may become stronger and statistically significant in older cohorts, as victimized males engage in more unhealthy behaviors that can increase inflammation levels (Danese and Baldwin, 2017). Future studies are needed to replicate these findings in other young cohorts and to investigate the mechanisms underlying the sexually dimorphic effects.

Third, building on earlier preliminary work in humans (Rooks et al., 2012), we tested in our large, population-representative study whether the association between a prospective measure of childhood victimization and CRP was influenced by genetic liability to high CRP. We found that victimized young women had high CRP levels independent of latent genetic influence. On the one hand, our findings are consistent with results from experimental animal studies that described environmental effects of early life stress on inflammation levels (Cole et al., 2012, Delpech et al., 2016). On the other hand, our findings expand previous knowledge by showing that similar environmental effects can also be observed in human populations characterised by greater genetic heterogeneity, and, thus, strengthen causal inference in humans.

Our findings should be considered in the context of some limitations. First, in order to maximise the collection of biological specimens in this large cohort, CRP was assessed in dried blood spots and not in serum. However, several previous studies have shown that CRP levels in dried blood spots are a valid proxy for serum equivalent (McDade et al., 2004, Danese et al., 2011, Sugden et al., 2015). Second, we undertook the present study in a cohort of twins, who may not be representative of the population of singletons. However, the prevalence of childhood victimization in this study is similar to the prevalence described in other studies of singletons (Lereya et al., 2015) and CRP levels in this study are similar to levels described in other samples of a similar age (Copeland et al., 2014), suggesting that our sample is representative of the population of singletons. Third, our index of genetic liability to CRP was based on a latent, twin-based method rather than on measured differences in DNA sequence. However, the current DNA-based method, namely the polygenic risk score, only accounts for a small proportion of the variance in CRP (5%) (Dehghan et al., 2011), which is less than the proportion of variance captured by our latent method (13%). Finally, this analysis was based on a cross-sectional measure of CRP, and therefore we could not examine whether childhood victimization predicted altered trajectories of CRP across development. Although CRP levels were measured at age 12 in a subsample of this cohort (Danese et al., 2011), only 154 Study members have CRP data at ages 12 and 18, and we were thus underpowered to test for an association between childhood victimization and changes in CRP levels over time.

Our findings suggest that females may be vulnerable to the effect of childhood victimization on inflammation in young adulthood. As such, young women who experienced childhood victimization may benefit from early interventions targeting inflammation including promotion of physical activity (Gleeson et al., 2011), healthy diet (Giugliano et al., 2006), psychological therapy (Creswell et al., 2012, Irwin et al., 2015) and possibly anti-inflammatory medication. Experimental work is now needed to test whether reduction of inflammation levels in victimized young women may prevent the emergence of later clinical disorders.
