# Supplementary material for: Childhood victimization and inflammation in young adulthood: A genetically sensitive cohort study
Source: Brain Behav Immun. 2018 Jan;67:211–7. doi: 10.1016/j.bbi.2017.08.025 (PMC5710993; doi:10.1016/j.bbi.2017.08.025)
Supplement: Supplementary data [file mmc1.docx]

**Supplementary Material**

**Method**

*Assessment of childhood victimization*

We have previously reported evidence on the reliability and validity of our measurement of childhood victimization (Danese et al., 2016). Here we summarize the method. A team of interviewers visited each family at home when the twins reached ages 5, 7, 10 and 12 years. Each home-visit interview was guided by a series of questions in a booklet. Based on these interviews with the mothers, each interviewer coded in the booklet her initial impression of whether or not she thought a child had been maltreated. The interviewers also recorded notes about their experiences in the home, and if an interviewer was worried about a child, she met with the fieldwork coordinator to debrief. Sometimes, the Study had to make a referral to help a child. Codes, notes, and the fieldwork coordinator’s narratives from the debriefs have been saved over the years to create a dossier for each child with cumulative information about exposure to domestic violence between the mother and her partner; frequent bullying by peers; physical maltreatment by an adult; sexual abuse; emotional abuse and neglect; and physical neglect. All the component measures are outlined briefly below.

Physical domestic violence. Mothers reported about perpetration by and victimization of 12 forms of physical violence (e.g., slapping, hitting, kicking, strangling) from the Conflict Tactics Scale (Straus et al., 1990), on three assessment occasions during the child’s first decade of life (when the children were 5, 7, and 10 years of age). Reports of either perpetration or victimization constituted evidence of physical domestic violence. Families in which no physical violence took place were coded as 0 (55.2%); families in which physical violence took place on one occasion were coded as 1 (28.0%); and families in which physical violence took place on multiple occasions were coded as 2 (16.8%).

Bullying by peers. Experiences of victimization by bullies were assessed using both mothers’ and children’s reports. During the interview, the following standard definition of bullying was read out: ―Someone is being bullied when another child (a) says mean and hurtful things, makes fun, or calls a person mean and hurtful names; (b) completely ignores or excludes someone from their group of friends or leaves them out on purpose; (c) hits, kicks, or shoves a person, or locks them in a room; (d) tells lies or spreads rumors about them; and (e) other hurtful things like these. We call it bullying when these things happen often, and when it is difficult to make it stop. We do not call it bullying when it is done in a friendly or playful way. Mothers were interviewed when children were 7, 10, and 12 years old and asked whether either twin had been bullied by another child, responding never, yes, or frequently. We combined mothers’ reports at child age 7 and 10 to derive a measure of victimization during primary school. Mothers’ reports when the children were 12 years old indexed victimization during secondary school. During private interviews with the children when they were 12 years old, the children indicated whether they had been bullied by another child during primary or secondary school. When a mother or a child reported victimization, the interviewer asked them to describe what happened. Notes taken by the interviewers were later checked by an independent rater to verify that the events reported could be classified as instances of bullying operationally defined as evidence of (a) repeated harmful actions, (b) between children, and (c) where there is a power differential between the bully and the victim (Shakoor et al., 2011).

Although inter-rater reliability between mothers and children was only modest (kappa = 0.20–0.29), reports of victimization from both informants were similarly associated with children’s emotional and behavioral problems, suggesting that each informant provides a unique but meaningful perspective on bullying involvement (Shakoor et al., 2011). We thus combined mother and child reports of victimization to capture all instances of bullying victimization for primary and secondary school separately: reported as not victimized by both mother and child; reported by either mother or child as being occasionally victimized; and reported as being occasionally victimized by both informants or as frequently victimized by either mother or child or both. We then combined these primary and secondary school ratings to create a bullying victimization variable for the entire childhood period (5–12 years). Children who were never bullied in primary or secondary school or occasionally bullied during one of these time periods were coded as 0 (55.5%); children who were occasionally bullied during primary and secondary school, or frequently bullied during one of these time periods were coded as 1 (35.6%); and children who were frequently bullied at both primary and secondary school were coded as 2 (8.9%).

Physical and sexual harm by an adult. We assessed childhood physical and sexual harm in the E-Risk Study using an approach that resembles the process undertaken by child protection agencies. Essentially this is a two-stage process. In child protection, professionals such as teachers working with children typically raise concerns if they observe signs or symptoms or if they become aware of risk that children are victims of violence. When concerns are raised, child protection officers then review the concerns and evaluate them in the context of information previously gathered on that child or family in order to determine the likelihood that abuse has taken place. In the E-Risk Study, research workers visited the home in pairs, and were extensively trained to detect signs of abuse or neglect. Each time the two research workers visited a home, they interviewed the mother using a structured interview about child harm, tested the children, and observed the family environment using the Home Observation for Measurement of the Environment (HOME; Bradley & Caldwell, 1977). If either research- worker had any concerns, they flagged up the case for review. Immediately after each home visit, a review was performed if a family was flagged. In addition, at each wave, any family who had been flagged on a prior wave of the study was automatically reviewed again. The reviews were performed independently by at least 2 clinical psychologists or psychiatrists, and were based on comprehensive dossiers compiled across multiple home visits for each study member during the course of the ongoing longitudinal study. When the twins were aged 5, 7, 10 and 12 their mothers were interviewed about each twins’ experience of intentional harm by an adult. At age 5 we used the standardized clinical protocol from the MultiSite Child Development Project (Dodge et al., 1990, Lansford et al., 2002). At ages 7, 10, and 12 this interview was modified to expand its coverage of contexts for child harm. Interviews were designed to enhance mothers’ comfort with reporting valid child maltreatment information, while also meeting researchers’ responsibilities for referral under the U.K. Children Act. Specifically, mothers were asked whether either of their twins had been intentionally harmed (physically or sexually) by an adult or had contact with welfare agencies. If caregivers endorsed a question, research workers made extensive notes on what had happened, and indicated whether physical and/or psychological harm had occurred. Under the U.K. Children Act, our responsibility was to secure intervention if maltreatment was current and ongoing. Such intervention on behalf of E-Risk families was carried out with parental cooperation in all but one case. No families left the study following intervention. Over the years of data collection, the study developed a cumulative profile for each child, comprising the caregiver reports, recorded debriefings with research workers who had coded any indication of maltreatment at any of the successive home visits, recorded narratives of the successive caregiver interviews, and information from clinicians whenever the Study team made a child- protection referral. Each time we visited a home, the research workers flagged concerns, and if there was sufficient evidence to code definite harm then, we did so. If evidence only met the level of probable harm, we kept an ongoing concern list and if, at a later wave, there was continued evidence of probable harm, or new evidence, the code was upgraded to definite harm. The profiles were reviewed at the end of the age–12 phase by at least two clinical psychologists or psychiatrists. Inter-rater agreement between the coders was 90% of cases for whom maltreatment was identified (100% for cases of sexual abuse), and discrepantly coded cases were resolved by consensus review. These were coded as: 0 = no physical harm at any age; 1 = probable physical harm at any age; and 2 = definite physical harm at any age. There were 15.0% of children coded as probably being exposed to physical harm and 5.1% as definitely physically harmed by 12 years of age. There were 1.5% of the children coded as being exposed to sexual abuse.

Emotional abuse and neglect. These forms of maltreatment were coded from research workers’ narratives of home visit at ages 5, 7, 10, 12. We coded quite severe examples of parental behavior observed. For example, a mother who had schizophrenia screamed and swore at the children throughout the home visit. As another example, a father who was drunk during the home visit repeatedly spoke abusively to the children in front of the research workers. We found that coders could not empirically separate emotional abuse and emotional neglect in a reliable way and thus such experiences were coded together as emotional abuse/neglect. Inter-rater agreement between the coders exceeded 85% for cases with such emotional abuse/neglect, and discrepant cases were resolved by consensus review. Children with no evidence of emotional abuse/neglect were coded as 0 (88.3%),

those where there was some indication of emotionally inappropriate/potentially abusive behavior were coded as 1 (8.7%), and where there was evidence of severe emotional abuse/neglect the children were coded as 2 (3%).

Physical neglect. The cumulative observations of the physical state of the home environment documented by the research workers during home visits to the twins at ages 5, 7, 10 and 12 were reviewed by two raters for evidence of physical neglect. This was defined as any sign that the caretaker was not providing a safe, sanitary, or healthy environment for the child. This included the child not having proper clothing or food, as well as grossly unsanitary home environments. (However, this did not include a family living in a crime-ridden neighborhood for economic reasons.) Inter-rater agreement between the coders was 85%, and discrepantly coded cases were resolved by consensus review. Children with no evidence of physical neglect were coded as 0 (90.9%), those for whom there was an indication of minor physical neglect were coded as 1 (7.1%), and where there was evidence of severe physical neglect the children were coded as 2 (2.0%).

**Results**

Supplementary Table 1. Sex differences in exposure to different types of severe or frequent childhood victimization. Results indicate risk of victimization in females compared to males.

| Childhood victimization type | Odds ratio (95% CI) | p-value |
| --- | --- | --- |
| Poly-victimization: 2+ types (N=110) | 0.54 (0.32;0.92) | 0.022 |
| Domestic violence (N=300) | 0.85 (0.60;1.21) | 0.38 |
| Bullying victimization (N=149) | 0.83 (0.56;1.23) | 0.36 |
| Physical maltreatment (N=97) | 0.47 (0.27;0.82) | 0.008 |
| Sexual abuse (N=14) | 3.52 (0.68;18.12) | 0.13 |
| Emotional abuse and neglect (N=53) | 0.44 (0.20;0.98) | 0.045 |
| Physical neglect (N=32) | 0.95 (0.35;2.55) | 0.92 |

References:

Bradley, R. H. & Caldwell, B. M. 1977. Home observation for measurement of the environment: a validation study of screening efficiency. *American Journal of Mental Deficiency,* 81**,** 417-420.

Danese, A., Moffitt, T. E., Arseneault, L., Bleiberg, B. A., Dinardo, P. B., Gandelman, S. B., Houts, R., Ambler, A., Fisher, H. L. & Poulton, R. 2016. The origins of cognitive deficits in victimized children: implications for neuroscientists and clinicians. *American Journal of Psychiatry,* 174**,** 349-361.

Dodge, K. A., Bates, J. E. & Pettit, G. S. 1990. Mechanisms in the cycle of violence. *Science,* 250**,** 1678-1683.

Lansford, J. E., Dodge, K. A., Pettit, G. S., Bates, J. E., Crozier, J. & Kaplow, J. 2002. A 12-year prospective study of the long-term effects of early child physical maltreatment on psychological, behavioral, and academic problems in adolescence. *Archives of Pediatrics & Adolescent Medicine,* 156**,** 824-830.

Shakoor, S., Jaffee, S. R., Andreou, P., Bowes, L., Ambler, A. P., Caspi, A., Moffitt, T. E. & Arseneault, L. 2011. Mothers and children as informants of bullying victimization: results from an epidemiological cohort of children. *Journal of Abnormal Child Psychology,* 39**,** 379-387.

Straus, M. A., Gelles, R. J. & Smith, C. 1990. *Physical violence in American families: Risk factors and adaptations to violence in 8,145 families*, Transaction Publishers New Brunswick, NJ.
